# CaMKII is a modulator in neurodegenerative diseases and mediates the effect of androgen on synaptic protein PSD95

**DOI:** 10.3389/fgene.2022.959360

**Published:** 2022-08-04

**Authors:** Shixiong Mi, Huan Chen, Peijing Lin, Peiyuan Kang, Dan Qiao, Bohan Zhang, Zhao Wang, Jingbao Zhang, Xiangting Hu, Chang Wang, Huixian Cui, Sha Li

**Affiliations:** ^1^ Department of Anatomy, Hebei Medical University, Shijiazhuang, China; ^2^ Neuroscience Research Center, Hebei Medical University, Shijiazhuang, China; ^3^ Hebei Key Laboratory of Neurodegenerative Disease Mechanism, Shijiazhuang, China; ^4^ School of Food Science and Engineering, South China University of Technology, Guangzhou, China; ^5^ Clinical Medicine, Hebei Medical University, Shijiazhuang, China; ^6^ Basic Medicine, Hebei Medical University, Shijiazhuang, China; ^7^ The Key Laboratory of Neural and Vascular Biology, Ministry of Education, Hebei Medical University, Shijiazhuang, China

**Keywords:** CaMKII, semantic similarity, protein interaction, androgen, Ca^2+^, PSD95

## Abstract

Androgens rapidly regulate synaptic plasticity in hippocampal neurones, but the underlying mechanisms remain unclear. In this study, we carried out a comprehensive bioinformatics analysis of functional similarities between androgen receptor (AR) and the synaptic protein postsynaptic density 95 (PSD95) to evaluate the effect. Using different measurements and thresholds, we obtained consistent results illustrating that the two proteins were significantly involved in similar pathways. We further identified CaMKII plays a critical role in mediating the rapid effect of androgen and promoting the expression of PSD95. We used mouse hippocampal neurone HT22 cells as a cell model to investigate the effect of testosterone (T) on intracellular Ca^2+^ levels and the mechanism. Calcium imaging experiments showed that intracellular Ca^2+^ increased to a peak due to calcium influx in the extracellular fluid through L-type and N-type voltage-gated calcium channels when HT22 cells were treated with 100 nM T for 20 min. Subsequently, we investigated whether the Ca^2+^/CaMKII signaling pathway mediates the rapid effect of T, promoting the expression of the synaptic protein PSD95. Immunofluorescence cytochemical staining and western blotting results showed that T promoted CaMKII phosphorylation by rapidly increasing extracellular Ca^2+^ influx, thus increasing PSD95 expression. This study demonstrated that CaMKII acts as a mediator assisting androgen which regulates the synaptic protein PSD95Also, it provides evidence for the neuroprotective mechanisms of androgens in synaptic plasticity and reveals the gated and pharmacological mechanisms of the voltage-gated Ca^2+^ channel family for androgen replacement therapy.

## 1 Introduction

According to the classical theory of steroid action, androgens enter the cell through the cell membrane and bind to the androgen receptor (AR) to form a complex. The complex then acts on specific DNA androgen reaction elements, initiating and regulating the transcription of related genes and affecting mRNA expression and protein synthesis (Gao et al., 2005; Jin et al., 2014). This hormone-receptor transcription pattern takes a long time, often hours or days. With research progression, more and more pieces of evidence show that the biological effects of androgen on various tissues and cells are difficult to explain by genomic effects, as they manifest as rapid non-genomic effects independent of traditional gene transcription regulation (Gu et al., 2009; Zhang et al., 2019). Foradori et al. summarized non-genomic effect characteristics, including faster speed than genomic effect, usually completed in a few seconds or minutes, membrane mediation, involving embedded or associated membrane receptors or ion channels, and no activation of the direct transcription/translation mechanism (Foradori et al., 2008). This mechanism may change intracellular Ca^2+^ concentration in different ways, increase cell membrane fluidity, and activate the second messenger pathway.

Androgens play an important role in the regulation of synaptic plasticity in the hippocampus. In male mice, the density of dendritic spines in the hippocampal CA1 region decreased significantly after castration. Androgen supplementation increased dendritic spine density to the level observed in mice with intact gonads (Li et al., 2015). The effect of androgens on hippocampal synaptic plasticity does not depend on their conversion to estrogen, because aromatase inhibitors do not affect androgens (Hatanaka et al., 2015). In contrast, androgen antagonists significantly attenuated the effect of androgens on dendritic spines (Jia et al., 2016). These reports suggest that androgens play an important role in maintaining normal dendritic spine density in the hippocampus of male animals.

The mechanism by which androgen effects rapidly regulate synaptic plasticity remains unclear. Postsynaptic density (PSD) is a complex composed of signal transduction proteins, neurotransmitter receptors, and coupled scaffold proteins. Postsynaptic density 95 (PSD95) is an important scaffold protein, which is usually used as a marker protein of postsynaptic plasticity and is involved in synaptic plasticity regulation (Zhu et al., 2020). In this study, we systematically evaluated the functional similarity between AR and PSD95 using their interactors. After network analysis and differential analysis, calcium/calmodulin-dependent protein kinase II beta (CaMK2B), which encodes a subunit of CaMKII, was determined to be a candidate mediating androgen effects on PSD95. CaMKII is a multifunctional serine/threonine kinase that has critical roles in synaptic plasticity, learning, and memory. Ca^2+^ is an important secondary messenger in neurones. Many extracellular signals, such as neurotransmitters and hormones, regulate Ca^2+^ content after interacting with receptors on cell membranes. Ca^2+^ activates protein phosphatase and participates in synaptic plasticity regulation (Roche et al., 1993; Zhang et al., 2021). As a result, we also investigated the mechanism, by which androgens affect synaptic protein PSD95 in HT22 cells through the Ca^2+^-induced Ca^2+^/CaMKII signaling pathway, and provided evidence for the neuroprotective effect of androgens.

## 2 Materials and methods

### 2.1 Protein–protein interaction data

The protein–protein interaction data were collected from the STRING database (v11.5) (Szklarczyk et al., 2021). To obtain reliable results, only interactions with confidence score greater than the cutoffs of 0.95 and 0.9 were used for subsequent analysis. The interactions in this database are defined as a functional association, i.e., the two linked proteins are jointly implemented in a shared biological function. In other words, the linked proteins are not necessarily physically binding to each other. All the associations in STRING are derived from eight evidence channels, including curated databases, experimentally determined, gene neighborhood, gene fusions, gene co-occurrence, text mining, co-expression, and protein homology.

### 2.2 Semantic similarity calculation

The functional similarity of genes or proteins can be calculated by Gene Ontology (GO) (Carbon et al., 2009). Currently, five semantic similarity measurements were widely used to compute the similarity between two GO terms, four of which measure the similarity based on the annotation statistics of their common ancestor terms, including Resnik, Jiang, Lin, and Schlicker (Yu et al., 2010). The other one named Wong utilized the graph structure of GO. Each measurement has both strengths and weaknesses. To perform a comprehensive estimation, all these methods were used for semantic similarity calculation using the R package GOSemSim (Yu et al., 2010; Yu, 2020).

### 2.3 Monte Carlo simulation

Monte Carlo simulations are typically used to model the probability of different outcomes in a process that cannot easily be predicted due to the intervention of random variables (Pandey and Farmer, 2013). In this study, we utilized Monte Carlo simulations to calculate the *p*-value of semantic similarity between two sets of proteins by randomly picking up protein set(s) of the same size and calculating the random similarities. The *p*-value is defined as the ratio of random values that greater than the real value over the random times. Two random sampling categories were used, i.e., creating random protein sets 1,000 times for both real sets or only one set.

### 2.4 Cell culture

Mouse hippocampal neurone cell line HT22 cells were cultured in DMEM containing 10% fetal bovine serum (GIBCO, United States) and 1% penicillin/streptomycin at 37°C and 5% CO_2_. When the cells reached 80–90% confluence, they were harvested through treatment with a solution containing 0.25% trypsin and resuspended in appropriate culture plates.

### 2.5 Calcium imaging

Free cytosolic Ca^2+^ was measured via fluorescence imaging using the Ca^2+^ indicator dye, Fluo-4 AM. A 5 mM stock solution of Fluo-4 AM (Bioworld Technology, United States) and 20% Pluronic F-127 (Thermo Fisher Scientific, United States) was prepared in DMSO (GIBCO, United States), diluted 1:1000 with HBSS and used within a week. When HT22 cells were 60–70% confluent, they were treated with 5 μM working solution Fluo-4 AM containing 0.02% Pluronic F-127 for 30 min at 37°C in the dark. Afterward, the Fluo-4 AM working solution was removed, and the cells were incubated for an additional 30 min in HBSS at 37 °C in the dark. Intracellular free calcium imaging was performed using confocal microscopy (FV1200, Olympus, Japan) at an excitation wavelength of 488 nm and an emission wavelength of 530 nm. Images of 800 × 800 pixels were acquired in XYZ scan mode using a ×60 objective (numerical aperture 1.35). The cells were perfused with HBSS for 5 min to obtain a basal fluorescence intensity level of intracellular Ca^2+^ (F0). This was followed by a further 55 min of treatment, including varying concentrations of testosterone (T; T0027, Tokyo Chemical Industry, Figure 4A), cyclopiazonic acid (CPA; C1530, Sigma-Aldrich), which inhibits intracellular calcium release, calcium-free extracellular fluid (NaCl 130 mM, KCl 3 mM, MgCl_2_ 4 mM, NaH_2_PO_4_ 1.25 mM, glucose 10 mM, NaHCO_3_ 26 mM), and EGTA (67425, Sigma-Aldrich), a chelating agent selective for Ca^2+^ (Figure 5A). The cells were also treated with amlodipine (A5605, Sigma-Aldrich), an L-type voltage-gated calcium channel blocker, and ω-Conotoxin-GVIA (CgTx; C9915, Sigma-Aldrich), an N-type voltage-gated Ca^2+^ channel blocker (Figure 6A), to obtain the real-time fluorescence signal intensity (F). Cells in the control group were treated with equal volume DMSO. Image-Pro Plus software (Media Cybernetics, United States) was used for further analysis. Changes in intracellular Ca^2+^ levels were expressed as F/F0.

### 2.6 L-type and N-type voltage-gated calcium channel blockade

HT22 cells were cultured on coverslips until 60–70% confluence. The cells were then cultured in the presence or absence of 5 μM amlodipine (A5605, Sigma-Aldrich) and 1 μM CgTx (C9915, Sigma-Aldrich) for 1 min before the start of T treatment, and those in the control group were treated with an equal volume of DMSO. The cells were incubated at 37°C for 20 min, and immunofluorescence cytochemistry and western blotting were performed.

### 2.7 CaMKII inhibition

HT22 cells were cultured on coverslips until 60–70% confluence. The cells were then cultured in the presence or absence of 5 μM KN-93 (HY-15465, MCE) for 2 h before treatment with T, and those in the control group were treated with equal volumes of DMSO. The cells were incubated at 37°C for 20 min, and immunofluorescence cytochemistry and western blotting were performed.

### 2.8 Immunofluorescence cytochemistry

HT22 cells plated on coverslips were fixed with 4% paraformaldehyde at room temperature for 15 min and sealed with 10% donkey serum at room temperature for 1 h. Afterward, the cells were incubated overnight at 4°C with the following primary antibodies: anti-PSD95 (ab18258, Abcam, United States) or anti-p-CaMKII (ab5683, Abcam, United States), incubated with donkey anti-rabbit fluorescent secondary antibody (A21206, Invitrogen, United States) for 2 h at room temperature in the dark, and counterstained with DAPI (Sigma, United States) for 10 min. Fluorescence images were obtained by using an inverted Olympus FV1200 confocal microscope system. The relative mean fluorescence intensity was measured using Image-Pro Plus software (Media Cybernetics).

### 2.9 Western blotting

RIPA lysis buffer containing phenylmethylsulfonyl fluoride was added to lyse HT22 cells, and proteins were extracted for quantification. Proteins were separated using SDS-PAGE and transferred onto a PVDF membrane. Then, the membranes were blocked with 5% milk for 2 h and incubated overnight at 4°C with the following primary antibodies: anti-PSD95 (ab18258, Abcam, United States), anti-p-CaMKII (ab5683, Abcam, United States), anti-CaMKII (ab52476, Abcam, United States), and anti-GAPDH (ab9485, Abcam, United States). Subsequently, they were incubated with DylightTM 800 goat anti-rabbit fluorescent secondary antibodies (611–145-002, Rockland, United States) in the dark for 2 h. Finally, imaging analysis was performed using an Odyssey imaging system (LICOR, United States). The relative expression of the target protein was calculated using the grey value of GADPH as a reference.

### 2.10 Statistical analysis

All statistical analyses were performed using SPSS 21.0 statistical software. Results are expressed as the means ± standard deviation (SD). Data sets were subjected to normality testing using the Shapiro-Wilk normality test. Data from multiple groups were tested for homogeneity of variance using Levene’s test. One-way ANOVA was performed for data with a normal distribution (*p* > 0.1) and homogeneity of variance (*p* > 0.1), and post-hoc multiple comparisons were performed using the *SNK-q* test. Differences were considered statistically significant at *p* < 0.05.

## 3 Results

### 3.1 Evaluation of functional similarity between AR and PSD95

**FIGURE 1:**
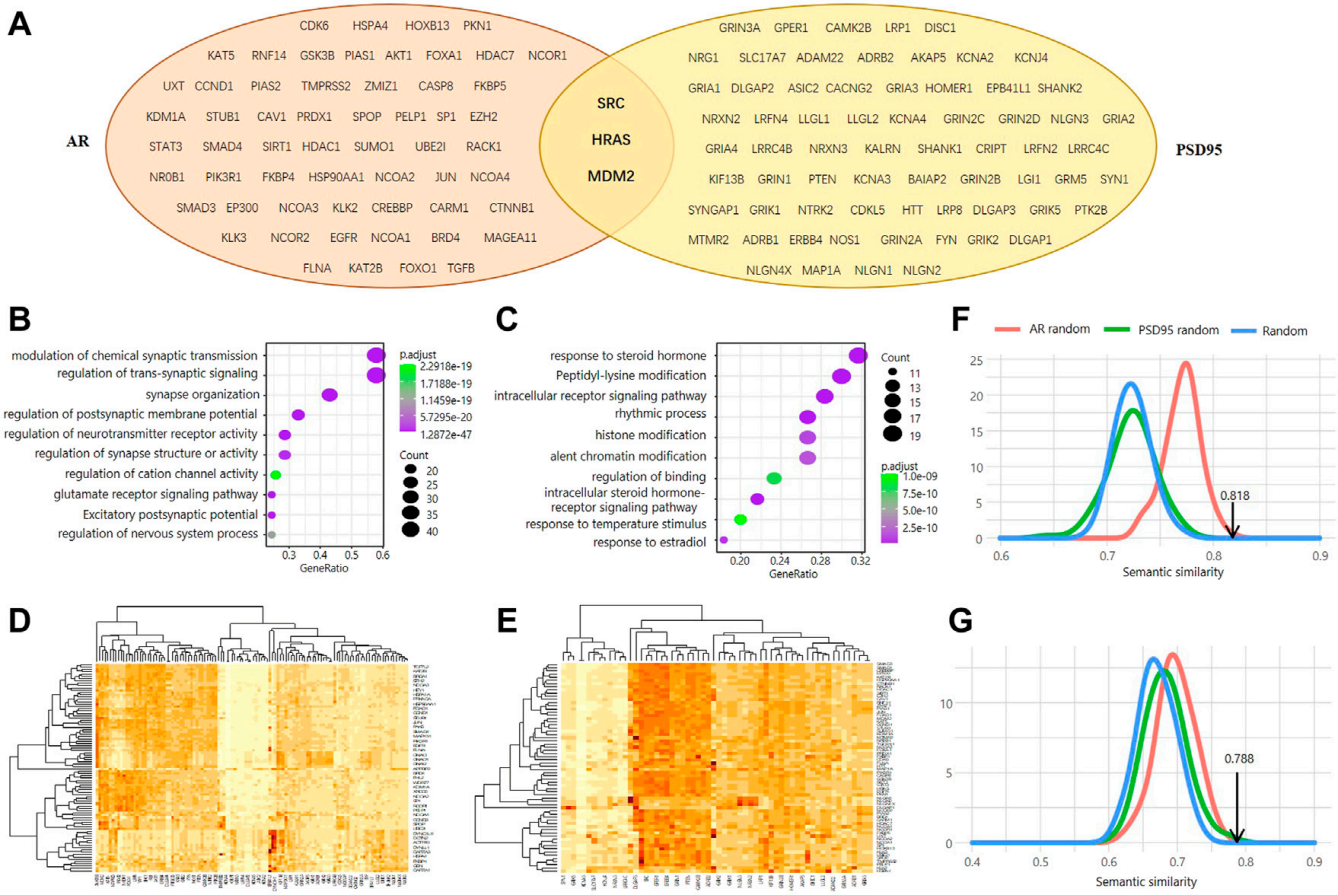
Genomic investigation of the association between AR and PSD95. **(A)** Venn diagram of the interactors between AR and PSD95. **(B,C)** Functional enrichment analysis of the PSD95 interactors **(B)** and AR interactors **(C)**. **(D,E)** Heatmap showing the semantic similarity between AR interactors and PSD95 interactors. Two thresholds of interaction confidence score, 0.9 **(D)** and 0.95 **(E)**, were used, respectively. **(F,G)** Distribution of the simulated SS scores calculated using wang. Three simulation methods were used and colored in red, green, and blue, respectively. Two thresholds of interaction confidence score, 0.9 **(F)** and 0.95 **(G)**, were used, respectively.

Using a strict threshold of 0.95 based on the STRING database, we determined 58 AR interactors and 68 PSD95 interactors with high confidence (Figure 1A). Three proteins were shared by both, including Proto-Oncogene Tyrosine-Protein Kinase Src (SRC), GTPase HRas (HRAS), and MDM2 proto-oncogene (MDM2). As expected, PSD95 interactors were involved in biological processes of regulation of trans-synaptic signaling, synapse organization, regulation of cation channel activity, etc. (Figure 1B), while AR interactors were enriched in functions of response to a steroid hormone, regulation of binding, response to estradiol, etc. (Figure 1C). These results illustrate the main functions that PSD95 and AR are implemented in.

Furthermore, we evaluated the functional similarity between the AR interactors and PSD95 interactors to probe whether these two proteins are associated. We observed that the Semantic Similarity Score (SSS) between AR interactors and PSD95 interactors is 0.818 when using the Interaction Confidence Score (ICS) greater than 0.95 (Figure 1D). The SSS is 0.788 using the ICS threshold of 0.9 (Figure 1E). To assess the statistical significance of the SSS, we simulated the protein interaction data of AR and PSD95 in three ways and performed each type of simulation 1,000 times. Our results show that the real SSS is significantly higher than the simulated ones (Figures 1F,G). In other words, randomly picked proteins with the same group size cannot achieve semantic similarity as high as AR and PSD95, indicating a potential pathway existing between them.

**FIGURE 2:**
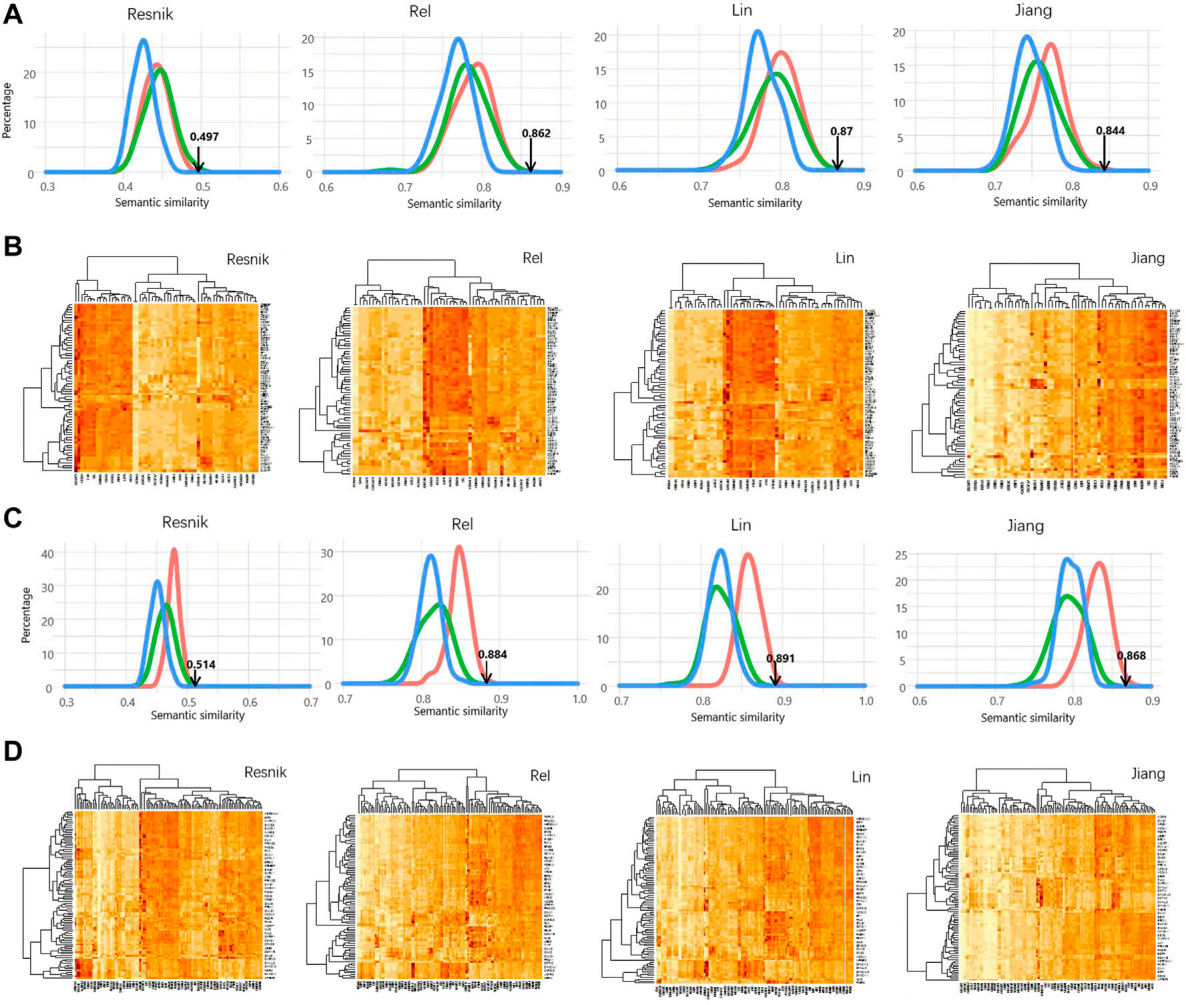
Significant high semantic similarity between AR interactors and PSD95 interactors. **(A)** Distribution of the simulated SS scores calculated using four different methods. The simulation methods were colored the same as Figure 1. **(B)** Heatmap showing the semantic similarity between AR interactors and PSD95 interactors using the four methods. Interaction confidence score of 0.9 was used in **(A,B)**. **(C)** Distribution of the simulated SS scores calculated using four different methods. **(D)** Heatmap showing the semantic similarity between AR interactors and PSD95 interactors using the four methods. Interaction confidence score of 0.95 was used in **(C,D)**.

**FIGURE 3:**
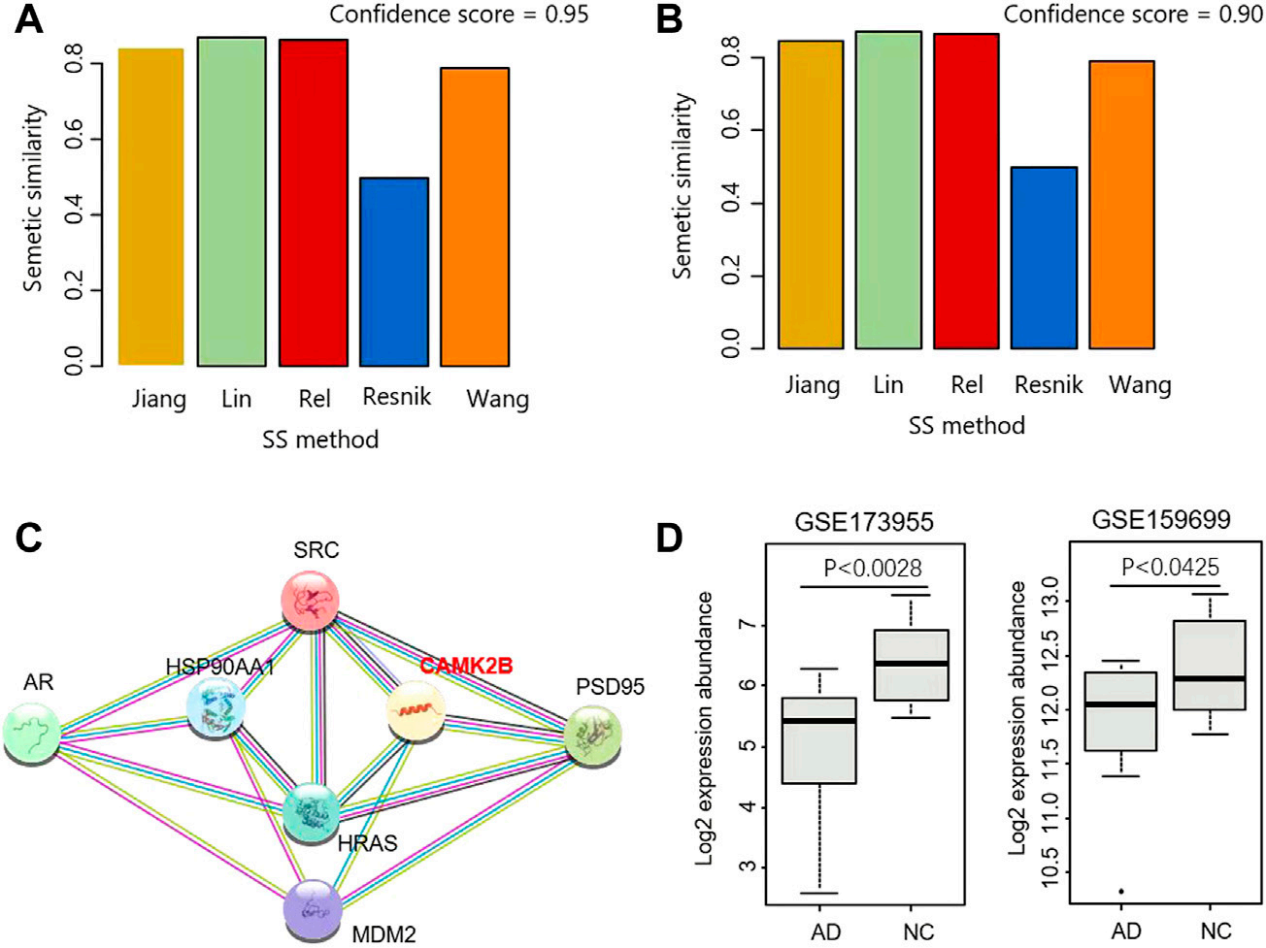
Identification of CaMKII as a mediator for androgen and PSD95. **(A,B)** SS scores were calculated by five methods based on the ICS threshold of 0.95 **(A)** and 0.9 **(B)**, respectively. **(C)** Interaction network including AR, PSD95 and their mediators. **(D)** CAMK2B were differentially expressed in two datasets GSE173955 and GSE159699.

For the above result, the SSS was computed using the Wang measurement. On top of that, four other methods were also used for calculating SSS, including Resnik, Rel, Lin, and Jiang. As shown in Figure 2, the real SSSs between AR interactors and PSD95 interactors are consistently high than the simulated scores, regardless of the semantic similarity measurements and the thresholds of ICS. Based on the ICS threshold of 0.95, the SSSs are 0.844, 0.87, 0.862, 0.497, and 0.788 for Jiang, Lin, Rel, Resnik, and Wong, respectively (Figure 3A). Based on the threshold of 0.90, the SSSs are 0.868, 0.891, 0.884, 0.514, and 0.818, for Jiang, Lin, Rel, Resnik, and Wong, respectively (Figure 3B).

### 3.2 Identification of CaMKII as a mediator for androgen and PSD95

Three proteins are the common interactors shared by AR and PSD95, i.e., SRC, HRAS, and MDM2 (Figure 1A). We further explored the proteins closely linking the three common interactors and found that CaMK2B is a hub connecting PSD95, SRC, HRAS, and MDM2 (Figure 3C). The product of CaMK2B belongs to the Ca^2+^/calmodulin-dependent protein kinase subfamily. Ca^2+^/calmodulin-dependent protein kinase that functions autonomously after Ca^2+^/calmodulin-binding and autophosphorylation, and is involved in the dendritic spine and synapse formation, neuronal plasticity, and regulation of sarcoplasmic reticulum Ca^2+^ transport in skeletal muscle. In neurones, it plays an essential structural role in the reorganization of the actin cytoskeleton during plasticity by binding and bundling actin filaments in a kinase-independent manner. In consequence, we performed the differential analysis of CaMK2B in two Alzheimer’s Disease RNA-sequencing (RNA-seq) datasets GSE173955 and GSE159699, and observed that CaMK2B was significantly differentially expressed in the two datasets (*p* < 0.0028 in GSE173955, *p* < 0.0425 in GSE159699, Wilcoxon ranksum test) (Figure 3D). Therefore, we identified CaMKII as a candidate regulator mediating the effect of androgen on PSD95 in neurodegenerative diseases.

### 3.3 Testosterone promotes extracellular Ca^2+^ influx through voltage-gated Ca^2+^ channels

**FIGURE 4:**
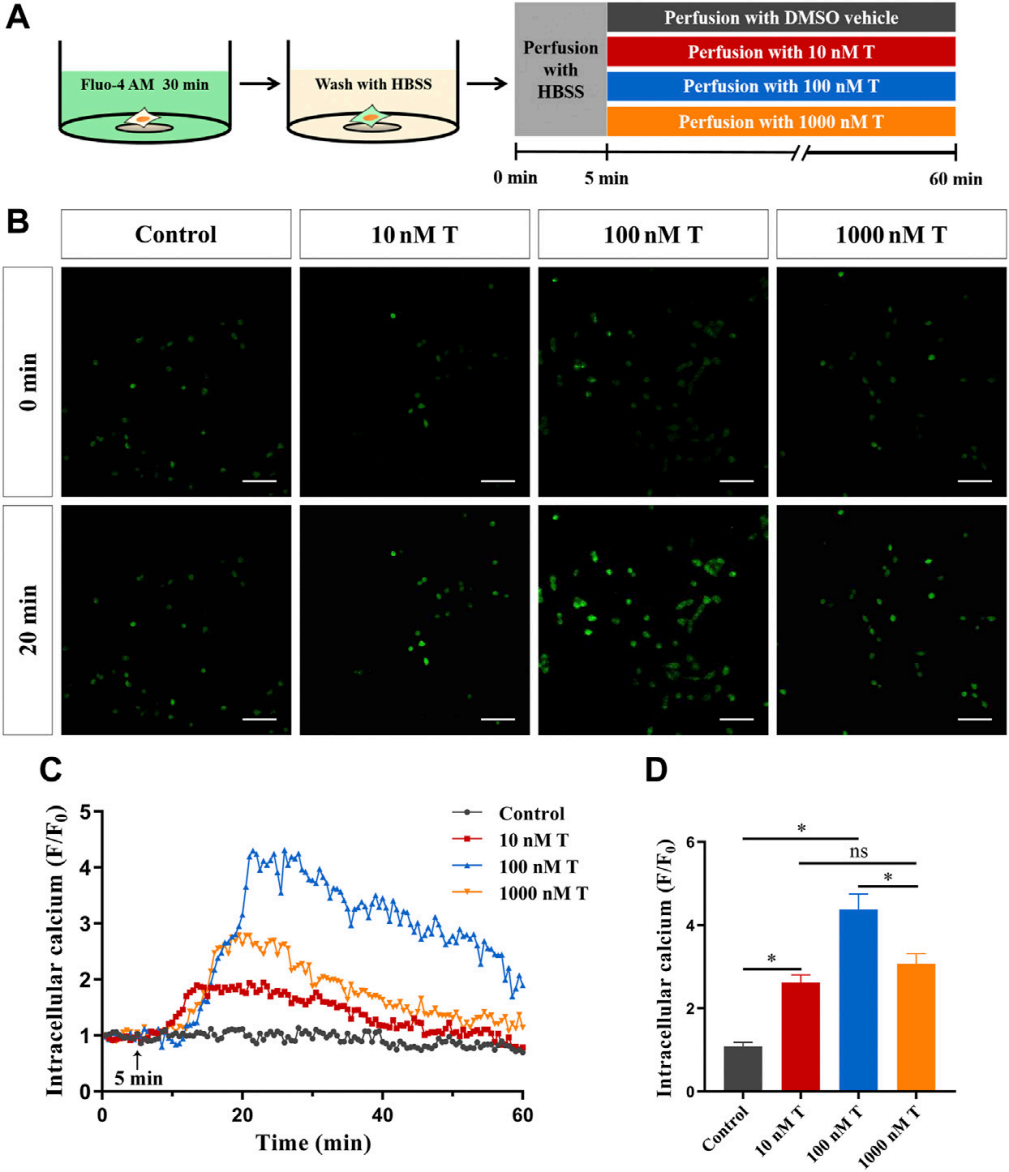
Rapid effects of T on calcium in HT22 cells. **(A)** Schematic diagram of calcium imaging in the T dose-response experiment in HT22 cells labeled with Fluo-4AM. **(B)** Fluorescence images of T dose-response experiment in Fluo-4AM-labelled HT22 cells. Scale bars = 50 μm. **(C)** Representative traces showing the changes in Ca^2+^ (F/F0) induced by different doses of T. Black arrows indicate when HT22 cells were treated. **(D)** Statistical graph showing changes in Ca^2+^ (F/F0) induced by different doses of T (ns: non-significant; *p < 0.05).

To assess the effect of T on Ca^2+^ concentration in HT22 cells, Fluo-4AM, a calcium indicator, was used to monitor intracellular calcium signals. The calcium signal of HT22 cells in the T treatment group increased significantly and reached its peak at 20 min, whereas DMSO did not cause fluorescence changes in the cells (Figure 4B). As the peak value of the intracellular calcium signal caused by 100 nM T increased significantly, compared with other concentrations (Figures 4B–D), we have chosen this T concentration for subsequent experiments.

**FIGURE 5:**
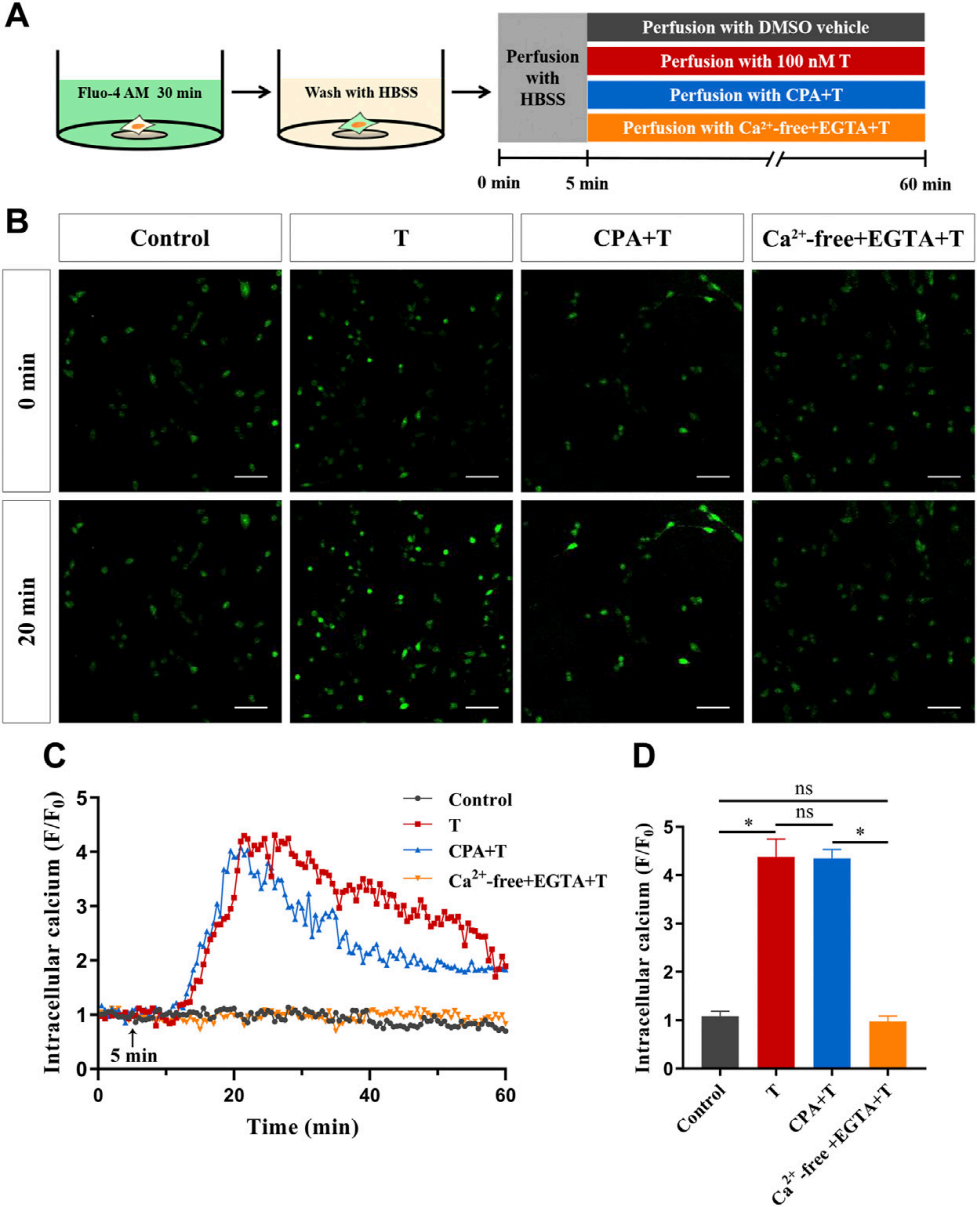
The source of increased intracellular Ca^2+^ induced by T in HT22 cells. **(A)** Schematic diagram of calcium imaging in the calcium source experiment in HT22 cells labeled with Fluo-4AM. **(B)** Fluorescence images of the calcium source in Fluo-4AM-labeled HT22 cells. Scale bars = 50 μm. **(C)** Representative traces showing changes in Ca^2+^ (F/F0) induced by T when HT22 cells were cultured in calcium-containing and calcium-free extracellular fluids. The black arrow indicates when the HT22 cells were treated. **(D)** Statistical graph showing the changes in Ca^2+^ (F/F0) induced by T when HT22 cells were cultured in calcium-containing and calcium-free extracellular fluids (ns: non-significant; *p < 0.05).

Next, to investigate the source of Ca^2+^, HT22 cells were treated with 100 nM T after blocking the intracellular calcium pool and using a calcium-free extracellular fluid. Calcium imaging results after endoplasmic reticulum calcium ATPase was inhibited with 5 μM cyclopiazonic acid showed that intracellular Ca^2+^ increased rapidly after T administration, and the degree of increase did not change significantly, compared with the T treatment group. With calcium-free extracellular fluid, the phenomenon of T-induced intracellular Ca^2+^ increase disappeared, indicating that the increase of intracellular Ca^2+^ mainly comes from extracellular calcium influx, rather than a release from the intracellular calcium pool (Figures 5B–D).

**FIGURE 6:**
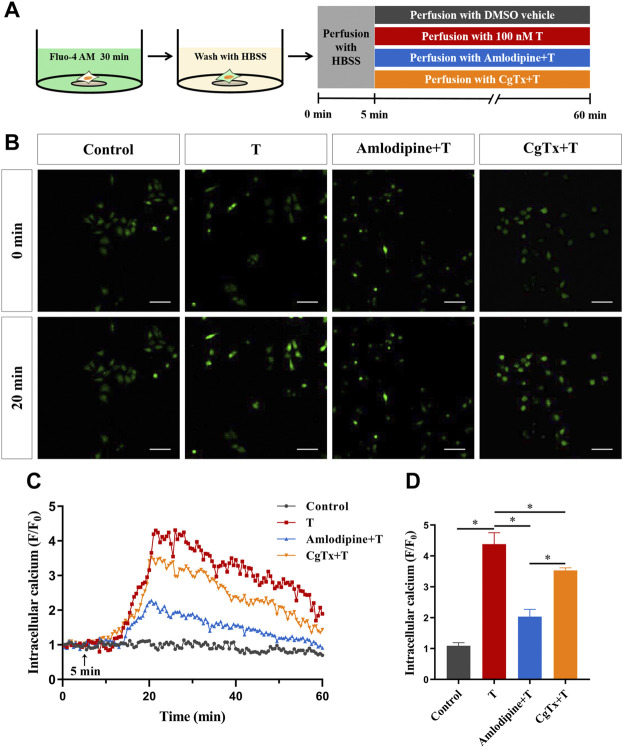
Voltage-gated calcium channels are involved in the T-induced increase of Ca^2+^ in HT22 cells. **(A)** Schematic diagram of calcium imaging in the voltage-gated calcium channel experiment in HT22 cells labeled with Fluo-4AM. **(B)** Fluorescence images of the voltage-gated calcium channel experiment in Fluo-4AM-labeled HT22 cells. Scale bars = 50 μm. **(C)** Representative traces showing changes in Ca^2+^ (F/F0) induced by T in HT22 cells treated with amlodipine and CgTx. The black arrow indicates when the HT22 cells were treated. **(D)** Statistical graph showing the T-induced changes in Ca^2+^ (F/F0) in HT22 cells treated with amlodipine and CgTx (ns: non-significant; *p < 0.05).

To clarify the pathway of extracellular Ca^2+^ influx, we investigated whether voltage-gated Ca^2+^ channels are involved in the T-induced increase in intracellular Ca^2+^ levels in HT22 cells. Calcium imaging results showed that T-induced F/F0 increase rates decreased significantly after administration of the L-type calcium channel blocker amlodipine (5 μM) or N-type calcium channel blocker CgTx (1 μM), compared with T alone. This suggested that voltage-gated Ca^2+^ channels, especially L-type calcium channels, are involved in T-induced increase in intracellular Ca^2+^ levels (Figures 6B–D).

### 3.4 Ca^2+^/CaMKII mediates the rapid effect of androgen on synaptic protein PSD95 in HT22 cells

**FIGURE 7:**
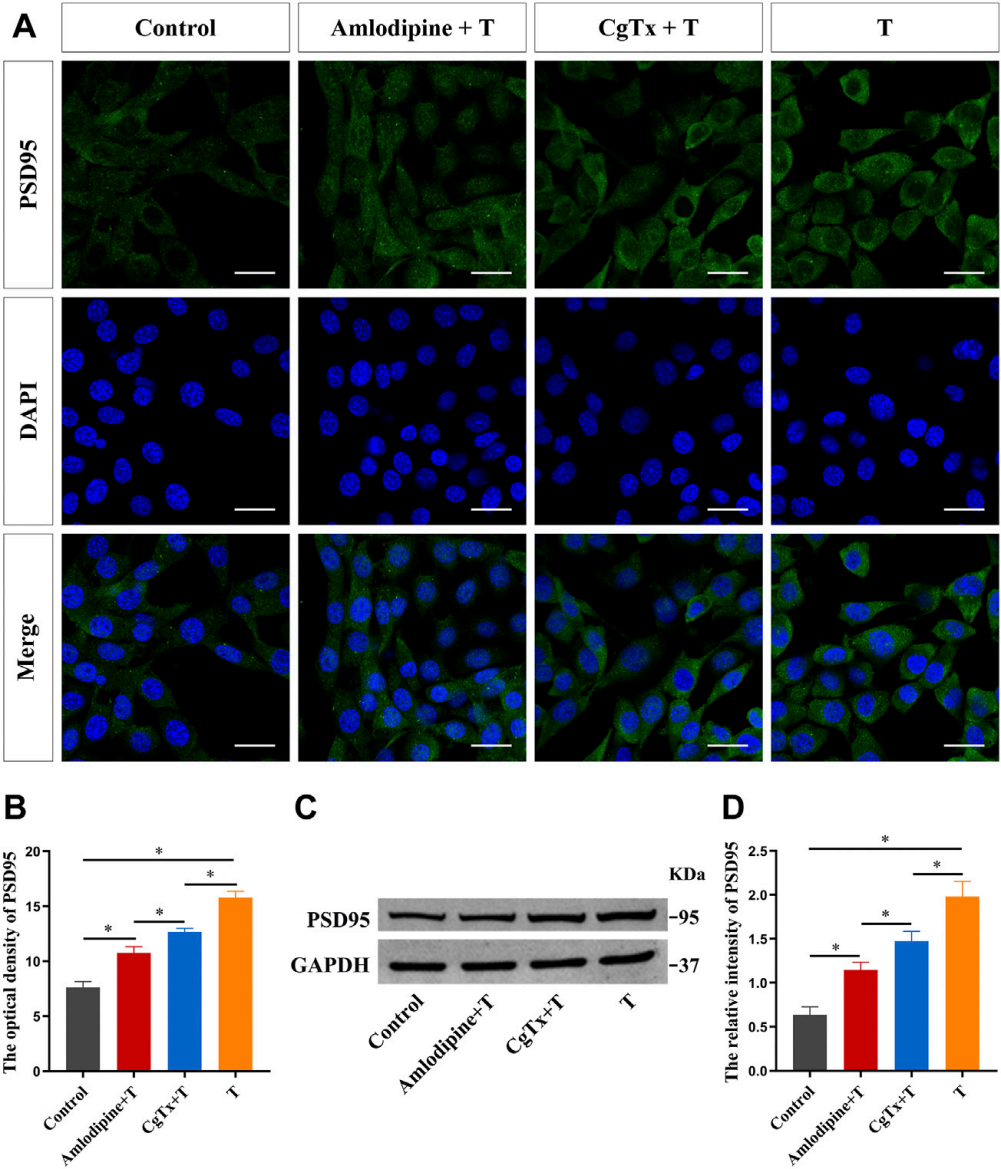
Voltage-gated calcium channels are involved in T-induced increase of synaptic protein PSD95 in HT22 cells. **(A)** Immunofluorescence cytochemistry for PSD95 expression in HT22 cells pretreated with T, amlodipine, or CgTx. **(B)** Statistical analysis of immunofluorescence cytochemistry for PSD95 expression in HT22 cells pre-treated with T, amlodipine, or CgTx. Scale bars = 20 μm. **(C)** Western blotting for PSD95 expression in HT22 cells pre-treated with T, amlodipine, or CgTx. **(D)** Statistical graph of western blot analysis of PSD95 expression in HT22 cells pre-treated with T, amlodipine, or CgTx (*p < 0.05).

To determine whether the Ca^2+^/CaMKII signaling pathway is involved in the regulation of synaptic protein PSD95 in HT22 cells, we conducted a series of experiments. First, we inhibited the L- and N-type voltage-gated Ca^2+^ channels to observe the effect of T on PSD95 protein expression. Immunofluorescence staining showed that the fluorescence intensity of the PSD95 protein in the T group was significantly higher than that in the control group. Pre-administration of amlodipine and CgTx efficiently inhibited the enhanced effect of T on PSD95 protein fluorescence intensity (Figures 7A,B). Western blotting results were consistent with immunofluorescence staining results. Amlodipine and CgTx inhibited PSD95 protein upregulation (Figures 7C,D).

**FIGURE 8:**
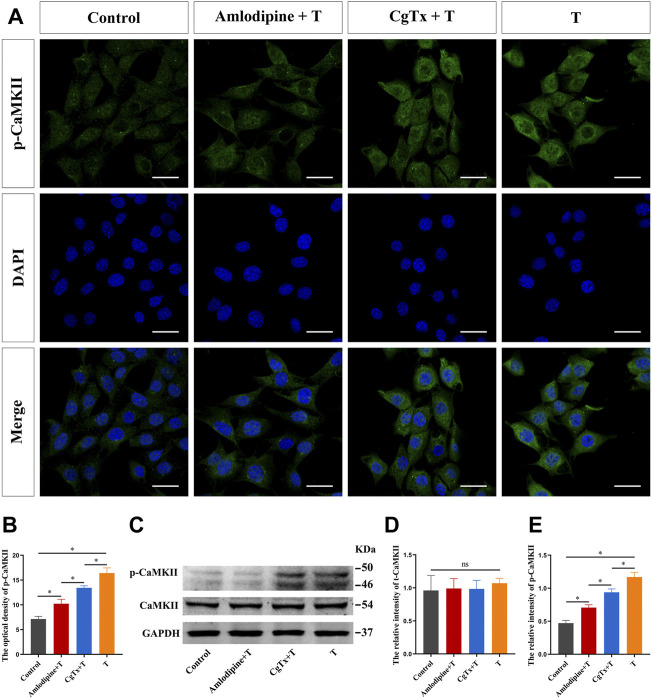
Voltage-gated calcium channels are involved in T-induced increase of CaMKII protein in HT22 cells. **(A)** Immunofluorescence cytochemistry for p-CaMKII expression in HT22 cells pre-treated with T, amlodipine, or CgTx. **(B)** Statistical analysis of immunofluorescence cytochemistry for p-CaMKII expression in HT22 cells pre-treated with T, amlodipine, or CgTx. Scale bars = 20 μm. **(C)** Western blotting for CaMKII and p-CaMKII protein expression in HT22 cells pre-treated with T, amlodipine, or CgTx. **(D,E)** Statistical graph of western blot analysis of CaMKII and p-CaMKII protein expression in HT22 cells pre-treated with T, amlodipine, or CgTx (*p < 0.05).

Subsequently, we inhibited L- and N-type voltage-gated Ca^2+^ channels to observe the effect of T on CaMKII and p-CaMKII protein expression in HT22 cells. Immunofluorescence staining showed that the fluorescence intensity of the p-CaMKII protein in the T group was significantly higher than that in the control group. Pre-administration of amlodipine and CgTx significantly inhibited the enhancing effect of T on p-CaMKII protein fluorescence intensity (Figures 8A,B). Western blotting results were consistent with immunofluorescence staining results. Amlodipine and CgTx inhibited the p-CaMKII upregulation by T. There was no significant difference in total CaMKII protein expression between all groups (Figures 8C–E).

**FIGURE 9:**
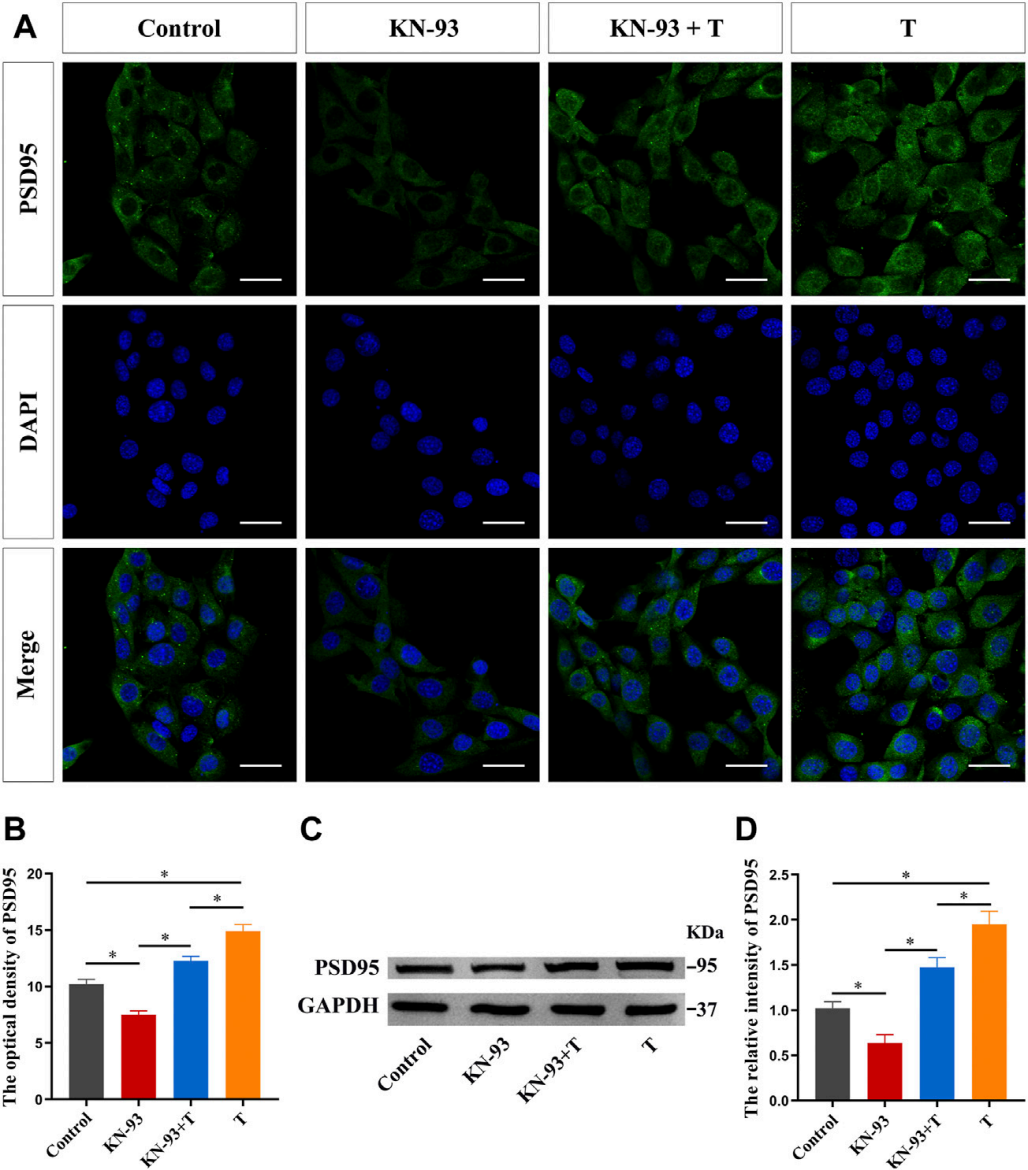
CaMKII protein is involved in the T-induced increase of synaptic protein PSD95 in HT22 cells. **(A)** Immunofluorescence cytochemistry of PSD95 expression in HT22 cells pre-treated with T or KN-93. **(B)** Statistical graph of immunofluorescence cytochemistry for PSD95 expression in HT22 cells pre-treated with T or KN-93. Scale bars = 20 μm. **(C)** Western blotting for PSD95 expression in HT22 cells pre-treated with T or KN-93. **(D)** Statistical graph of western blot analysis of PSD95 expression in HT22 cells pre-treated with T or KN-93 (*p < 0.05).

Finally, we examined the effect of T on PSD95 protein expression after CaMKII inhibition in HT22 cells. Immunofluorescence staining results showed that pre-treatment with KN-93 inhibited PSD95 protein fluorescence intensity, as well as the enhanced effect of T on PSD95 protein fluorescence intensity (Figures 9A,B). Western blotting results were consistent with immunofluorescence staining results. KN-93 inhibited PSD95 protein upregulation (Figures 9C,D).

## 4 Discussion

Androgens can affect the structure and function of the hippocampus, and subsequently affect learning and memory, as well as spirit, emotion, and mood. For example, androgen level changes show different effects in rodent experimental animal models (Shao et al., 2020), non-human primate models (Wallen, 2005), human cognitive function (Zitzmann, 2006; Hamson et al., 2016), and other neurobehaviors, demonstrating a correlation between androgen level changes and the occurrence of neurodevelopmental disorders (Romano et al., 2016) and neurodegenerative diseases (Pike et al., 2008). Immunoelectron microscopy and other techniques have shown that ARs are widely expressed in hippocampal neurone nuclei, as well as in extranuclear sites, such as cell membranes, mitochondria, and synaptic vesicles (Tabori et al., 2005; Sarkey et al., 2008). Demonstrating the localization of these ARs in hippocampal neurones provides a morphological basis for the hippocampus as an androgen target organ. However, it is not fully understood how these ARs mediate androgen effects on hippocampal neurons.

The rapid effects of androgens are thought to occur mainly through membrane regulatory mechanisms, including embedded and associated membrane receptors and ion channels (Zhang et al., 2019; Lorigo et al., 2020). These effects are observed even if androgen binding to membrane sites cannot enter the cytoplasm, or if androgen binding to receptors cannot be transferred to the nucleus. Acute hippocampal slices of adult male rats incubated with physiological concentrations of dihydrotestosterone (DHT) or T significantly increased dendritic spine density in the CA1 region after 2 h (Hatanaka et al., 2009; Hatanaka et al., 2015). However, when analyzing the head diameter of dendritic spines, the effects of DHT and T were different. After acute hippocampal slices were incubated in DHT for 2 h, medium (0.4–0.5 μm) and large (0.5–1.0 μm) head dendritic spine densities in the CA1 region increased significantly, but small (0.2–0.4 μm) head dendritic spine density did not change significantly. After acute hippocampal slices were incubated in T for 2 h, the small head dendritic spine density increased significantly, but the densities of medium and large head dendritic spines did not change significantly (Hatanaka et al., 2015). Although T can be converted to estrogen by aromatase, letrozole, an aromatase inhibitor, did not inhibit the effect of T on the dendritic spines of hippocampal neurones (Hatanaka et al., 2015). Furthermore, electron microscopic analysis of dendritic spine density showed that the AR antagonist flutamide did not inhibit the increase in dendritic spine density induced by DHT (MacLusky et al., 2006; Hajszan et al., 2008). Our previous study found that HT22 cells have androgen membrane-binding sites and that T-BSA could affect the expression of synaptic protein PSD95 through rapid effects (Zhang et al., 2019).

At present, the mechanism of androgen rapid effects on dendritic spines and synaptic proteins in hippocampal neurones is unclear. The calcium regulatory mechanism is a rapid response that occurs within seconds to minutes and is presumably mediated by androgen interaction with binding sites on the cell surface (Lieberherr and Grosse, 1994; Panagiotopoulos et al., 2021). This study investigated whether androgens could induce rapid changes in Ca^2+^ levels in HT22 cells. A Fluo4-AM calcium probe was used to label calcium ions in HT22 cells, and different concentrations of T were administered. Intracellular Ca^2+^ levels increased rapidly, and intracellular calcium fluctuation induced by T administration was observed using confocal laser microscopy. The change in intracellular Ca^2+^ was most obvious at 100 nM T, and the peak appeared 20 min after administration, indicating that 100 nM T significantly induced a rapid change in intracellular Ca^2+^ in HT22 cells. The effects of testosterone as a pharmacological agent acting on neuronal cells and calcium as a messenger ion are quite significant, and they may hold the potential for treat degenerative diseases.

As a ubiquitous secondary messenger, Ca^2+^ is essential for almost all life processes. Ca^2+^ signaling, an indicator of neural activity, plays an essential role in neural development (Toth et al., 2016), synaptic plasticity (Cavazzini et al., 2005), learning, and memory (Jeon et al., 2003). To investigate the source of intracellular Ca^2+^ increase, HT22 cells were treated with calcium-containing and calcium-free extracellular fluids. With calcium-free extracellular fluid, the increase in intracellular calcium ions induced by T disappeared, indicating that the increase mainly came from extracellular calcium influx, rather than intracellular calcium pool release. These results suggest that calcium-permeable ion channels in cell membranes play an important role in T-induced increase in intracellular calcium ions. We then administered the L- and N-type calcium channel blockers amlodipine and CgTx, respectively, in HT22 cells and found that T-induced intracellular Ca^2+^ increase was significantly inhibited. These results indicate that voltage-gated Ca^2+^ channels, especially L-type Ca^2+^ channels, participate in T-induced intracellular Ca^2+^ increase.

Androgens cause rapid external calcium influx and increase intracellular Ca^2+^, which provides the basis for Ca^2+^ to participate in physiological activities as a second messenger. Large amounts of Ca^2+^ enter cells, bind to calmodulin (CaM), activate CaMKII, and affect synaptic plasticity through a series of cascade reactions (Bayer et al., 2006). CaMKII is highly expressed in brain tissues, especially in the hippocampus, and it accounts for approximately 2% of the total protein content (Erondu and Kennedy, 1985). CaMKII can activate glutamate receptors, which alter neuronal excitability and synapse protein synthesis (Lisman et al., 2012). Changes in hippocampal synaptic plasticity caused by androgen deficiency include abnormal expression of synapse-associated proteins (Zhao et al., 2018), decreased dendritic spine density (Leranth et al., 2003), and decreased synaptic transmission efficiency (Cooke and Woolley, 2009). PSD95, a scaffold protein, is primarily located in the excitatory glutamic energy postsynaptic membrane (Delgado et al., 2020). PSD95 is a key protein that promotes synapse maturation and maintains the stability of dendritic spines (Ampuero et al., 2017). PSD95 regulates the number of synapses during development (Gilbert and Man, 2017) and plays an important role in synaptic plasticity. However, the mechanism underlying the rapid effect of T on the hippocampal synaptic protein PSD95 remains unclear.

In this study, immunofluorescence cytochemistry and western blotting were performed to determine whether T rapidly affects the expression of the synaptic protein PSD95 through the Ca^2+^/CaMKII pathway in HT22 cells for the first time. To begin with, we inhibited L- and N-type voltage-gated Ca^2+^ channels and observed that the T-induced upregulation of PSD95 was significantly inhibited, suggesting that the T-induced increase in PSD95 protein partially depended on calcium influx caused by voltage-gated Ca^2+^ channel opening. This study confirmed that T could rapidly activate CaMKII phosphorylation in HT22 cells, while inhibition of L- or N-type voltage-gated calcium channels weakened the activation of CaMKII. In the central nervous system, activation of NMDA receptors on the postsynaptic membrane leads to an increase in the local concentration of postsynaptic Ca^2+^, which binds to and activates CaM. Activated CaM activates Ca^2+^/CaM-dependent CaMKII, which plays an important role in synaptic plasticity, learning, and memory (Zhang et al., 2021). KN-93 is a CaMKII inhibitor, which reduces the expression level and activity of CaMKII, as well as the phosphorylation level of its phosphorylation site Thr^305^ (Munevar et al., 2008). In this study, we have observed for the first time that KN-93 can reduce the expression of the synaptic protein PSD95 in HT22 cells. In addition, administration of the CaMKII inhibitor KN-93 also significantly inhibited the T-induced upregulation of PSD95.

The results of this study showed that T promoted CaMKII phosphorylation by rapidly increasing the influx of external Ca^2+^ and upregulating the expression of PSD95. This study reveals the pharmacological mechanism of androgen replacement therapy mediated by the voltage-gated Ca^2+^ channel family, which contributes to a full understanding of the physiological role of androgens and provides evidence for further research on the neuroprotective mechanism of androgens.

## Data Availability

The original contributions presented in the study are included in the article/supplementary material; further inquiries can be directed to the corresponding authors.
